# Targeting Molecular Mechanisms of Obesity- and Type 2 Diabetes Mellitus-Induced Skeletal Muscle Atrophy with Nerve Growth Factor

**DOI:** 10.3390/ijms25084307

**Published:** 2024-04-13

**Authors:** Lauren Jun, Xiao-Wen Ding, Megan Robinson, Hassan Jafari, Emily Knight, Thangiah Geetha, Michael W. Greene, Jeganathan Ramesh Babu

**Affiliations:** 1 Department of Nutritional Sciences, Auburn University, Auburn, AL 36849, USA; 2 Boshell Metabolic Diseases and Diabetes Program, Auburn University, Auburn, AL 36849, USA

**Keywords:** obesity, diabetes, muscle atrophy, western diet, nerve growth factor

## Abstract

Skeletal muscle plays a critical role in metabolic diseases, such as obesity and type 2 diabetes mellitus (T2DM). Muscle atrophy, characterized by a decrease in muscle mass and function, occurs due to an imbalance between the rates of muscle protein synthesis and degradation. This study aimed to investigate the molecular mechanisms that lead to muscle atrophy in obese and T2DM mouse models. Additionally, the effect of nerve growth factor (NGF) on the protein synthesis and degradation pathways was examined. Male mice were divided into three groups: a control group that was fed a standard chow diet, and two experimental groups that were fed a Western diet. After 8 weeks, the diabetic group was injected with streptozotocin to induce T2DM. Each group was then further divided into NGF-treated or non-treated control group. In the gastrocnemius muscles of the Western diet group, increased expressions of myostatin, autophagy markers, and ubiquitin ligases were observed. Skeletal muscle tissue morphology indicated signs of muscle atrophy in both obese and diabetic mice. The NGF-treated group showed a prominent decrease in the protein levels of myostatin and autophagy markers. Furthermore, the NGF-treated group showed an increased Cyclin D1 level. Western diet-induced obesity and T2DM may be linked to muscle atrophy through upregulation of myostatin and subsequent increase in the ubiquitin and autophagy systems. Moreover, NGF treatment may improve muscle protein synthesis and cell cycling.

## 1. Introduction

Obesity is a pervasive metabolic disease affecting both adults and children alike, with a significantly increased prevalence in the world’s population over the past three decades [1]. A major contributor to this trend is the chronic overconsumption of Western diets, characterized by high levels of refined sugars, saturated fats, and processed foods. This SAD (standard American diet) pattern contributes to excess calorie intake, leading to abnormal accumulation of adipose tissue. Over time, the chronic energy balance state can exacerbate the development of metabolic and cardiovascular diseases. In particular, obese adults face an elevated risk of developing type 2 diabetes mellitus (T2DM), with approximately 89% of adults with T2DM being overweight or obese [2].

T2DM is a complex metabolic disorder characterized by hyperglycemia resulting from insulin resistance and pancreatic failure. Overweight and obesity, especially in cases with aberrant adipose tissue accumulation and the associated chronic low-grade inflammation, have been implicated in pancreatic β-cell dysfunction which contributes to insulin resistance [3]. As a result of prolonged insulin resistance, β-cells may not be able to meet the increased demand of insulin and thus lead to insufficient hepatic and peripheral glucose disposal, followed by higher circulating glucose levels and eventually to the development of T2DM [3]. Globally, nearly half a billion people in the world live with T2DM [4], causing a significant burden on the healthcare systems and individuals and their families. The link between obesity and the emergence of T2DM, therefore, highlights the urgent need for effective preventative strategies and interventions to lessen the health crises. Skeletal muscle plays a crucial role in the human body, accounting for up to 40% of total body mass. Not only does it power movement and energy metabolism, but it also plays a central role in regulating glucose levels and insulin sensitivity. In fact, skeletal muscle is responsible for more than 80% of glucose uptake following a meal [5]. However, in conditions such as T2DM, muscle insulin resistance can result in hyperglycemia. Therefore, maintenance of muscle mass and its function is important for overall health and metabolic balance [6].

The regulation of skeletal muscle mass involves an intricate balance between the rates of protein synthesis and degradation [7]. Muscle hypertrophy is typically characterized by increased protein synthesis and decreased protein degradation, whereas muscle atrophy is characterized by decreased protein synthesis and increased protein degradation [7]. These anabolic and catabolic processes are mediated by several endocrine, paracrine, and autocrine signaling pathways that influence muscle mass [7,8]. These signaling pathways help explain why energy states, physical mobility, and disease states like T2DM and obesity can significantly influence muscle mass [7,9].

Myostatin, also called growth differentiation factor 8 (GDF-8), is a myokine that belongs to the transforming growth factor (TGF)-β superfamily. Since its discovery, myostatin has been recognized as a potential mediator of muscle atrophy in various pathological conditions, including obesity [10] and T2DM [11]. Myostatin is, in fact, one of the primary negative regulators of muscle growth [12]. This molecule is abundantly expressed in skeletal muscles and activates Smad2 and Smad3, which form a complex with Smad4 and interact with various signaling pathways, regulating the transcription of genes associated with muscle protein synthesis and degradation [13]. For instance, previous studies have highlighted the action of myostatin in activating ubiquitin E3 ligases, MuRF1 and MAFbx (Atrogin-1) [14,15], and autophagy marker LC3 [15]. Therefore, inhibiting myostatin has been of great interest in preventing muscle atrophy caused by various metabolic conditions. To date, numerous myostatin-targeting strategies involving antibodies, myostatin pro-peptides, soluble receptors, and endogenous antagonists have been investigated [16]. However, as these therapeutic approaches differ in efficacy and safety, continuous investigation is warranted.

Neurotrophins are a class of trophic factors that play a significant role in regulating neuronal cell proliferation, differentiation, growth, survival, and apoptosis [17]. This family of neurotrophins includes nerve growth factor (NGF), brain-derived neurotrophic factor (BDNF), neurotrophin-3 (NT-3), and neurotrophin-4 (NT-4). It has been suggested that NGF is primarily expressed in the central nervous system, but recent evidence suggests that NGF and its receptors, tropomyosin receptor kinase A (TrkA) [18] and pan-neurotrophin receptor p75 (p75NTR), are also expressed in muscle cells [19,20]. Notably, myoblasts endogenously produce NGF and other neurotrophins in the course of developing new muscles, which can have an autocrine effect on proliferation, fusion into myotubes, and cell morphology [18]. In a study by Ruberti, et al. phenotypic knockout mice expressing transgenic antibody against NGF displayed severe muscular dystrophy [21], highlighting the importance of NGF in muscle maintenance. While previous studies have used muscle cell lines to observe the effect of NGF in muscle regeneration, further investigations are needed to explore the role of NGF in skeletal muscle tissue maintenance.

Thus, this study aims to investigate the molecular mechanisms that lead to muscle atrophy in obese and T2DM mouse models. Additionally, this study aims to explore the effect of NGF on protein synthesis and degradation pathways, providing a comprehensive understanding of its role in skeletal muscle maintenance and progression toward atrophy.

## 2. Results

### 2.1. Effects of High Fructose and Sucrose (HFS) Diet and Streptozotocin (STZ) Injections on Body Mass and Fasting Blood Glucose Level

**Figure 1:**
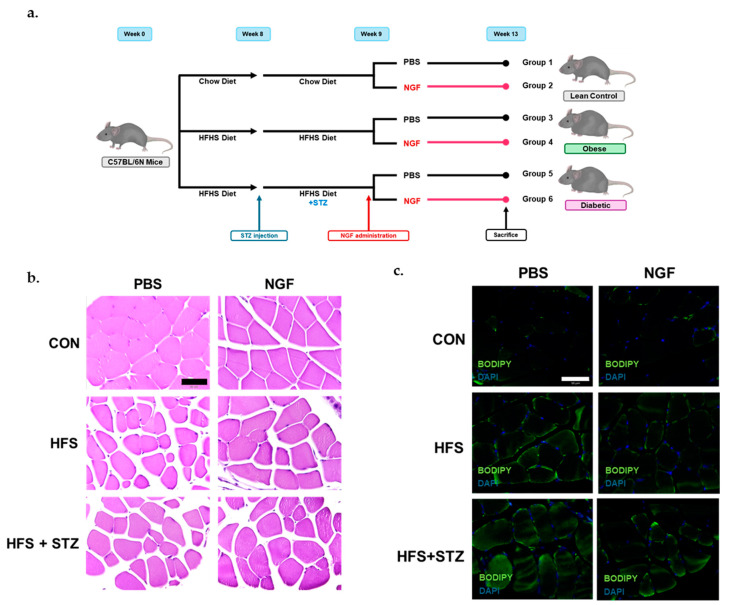
Skeletal muscle morphological characteristics in obese and T2DM mice. (**a**) Schematic of the animal diets. (**b**) Representative sections of gastrocnemius muscles stained with Hematoxylin and Eosin (H&E); 40×; scale bar 50 μm; *n* = 3 in each group. (**c**) Representative sections of gastrocnemius muscles stained with BODIPY^TM^ indicating intramyocellular lipid accumulation in the HFS and HFS + STZ groups; 40×; scale bar 50 μm; *n* = 3 in each group.

Figure 1a shows a schematic of the diet and treatment regimen. In brief, male C57BL/6N mice were fed either a normal chow diet or a Western diet for 13 weeks. At week 8, HFS + STZ and HFS + STZ + NGF groups were injected with a low dose of STZ to induce T2DM conditions. At week 9, NGF was administered intranasally to the mice in lean control (Ln) + NGF, HFS + NGF, HFS + STZ + NGF groups. Compared to the Ln control, HFS and HFS + STZ groups had a significantly higher body weight starting at week 2 and continued to diverge until the end of the study period. Furthermore, HFS + STZ and HFS + STZ + NGF groups had significantly higher fasting blood glucose levels (>200 mg/dL), before sacrifice, compared to the Ln control group, indicating the development of T2DM.

### 2.2. Skeletal Muscle Morphological Characteristics in Obese and T2DM Mice

To evaluate the skeletal muscle morphology of the mice, paraffin-embedded gastrocnemius muscle sections were stained with Hematoxylin and Eosin (Figure 1b). The visual observation of the tissue morphology of the lean control mice did not show any indication of muscle atrophy; however, HFS and HFS + STZ groups showed visual indications of muscle atrophy depicted by irregularly shaped fibers and separations between them (Figure 1b). In addition, BODIPY^TM^ staining was performed to observe the accumulation of intramyocellular lipids. The image of lipid staining showed an accumulation of lipid droplets around muscle fibers in the HFS and HFS + STZ groups (Figure 1c).

### 2.3. Obesity Increases Protein Level of Myostatin

**Figure 2:**
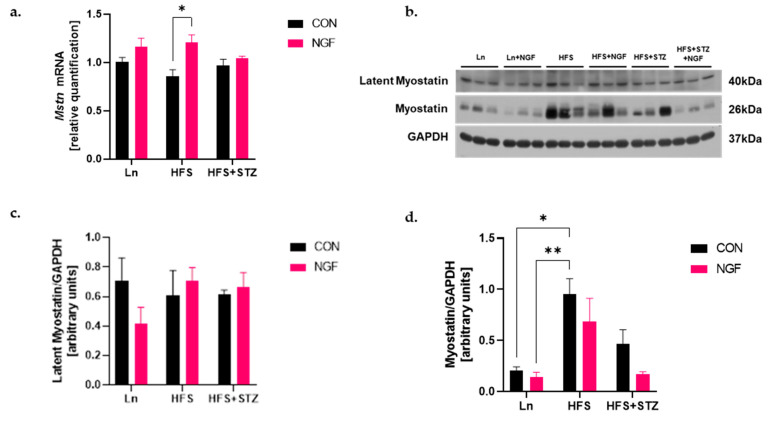
Obesity increases activation of myostatin and NGF prevents the action of myostatin. (**a**) RT-PCR results indicate a significant increase in the *Mstn* mRNA expression in HFS + NGF vs. HFS, * *p* < 0.05. (**b**) Western blot analysis of latency associated protein (LAP) myostatin complex (40 kDa) and active myostatin (26 kDa). (**c**) Densitometry of LAP myostatin levels. (**d**) Densitometry of active myostatin. A significantly increased level was observed in HFS vs. Ln and Ln + NGF, * *p* < 0.01 and ** *p* < 0.001, respectively. A significant decrease was observed in HFS vs. HFS + STZ. Data are shown as means ± SEM, *n* = 3 in each group.

Myostatin protein is synthesized and secreted from myoblasts, then processed to regulate myoblast growth and differentiation systemically [22]. Thus, secretion, as well as processing of myostatin protein, are critical during muscle development. To observe myostatin’s transcriptional and post-translational events, we analyzed the levels of *Mstn* mRNA and the levels of proteolytically processed N-terminal latency associated peptide (LAP) and C-terminal mature myostatin peptide, respectively. As a result, reverse transcriptase-polymerase chain reaction (RT-PCR) revealed that *Mstn* mRNA was significantly increased in the HFD + NGF group compared to the non-treated counterpart (Figure 2a, * *p* < 0.05, HFS + NGF vs. HFS). In the Western blot analysis using an antibody that recognizes both precursor and mature forms of myostatin, no appreciable differences in the levels of LAP were observed between groups (Figure 2c). However, the level of cleaved myostatin (26 kDa) was significantly higher in the HFS group compared to Ln control (Figure 2d, * *p* < 0.05 and ** *p* < 0.01 HFS vs. Ln and Ln + NGF, respectively). Furthermore, although insignificant, these increased levels of myostatin in both HFS and HFS + STZ groups were attenuated in the NGF-treated groups (Figure 2d).

### 2.4. NGF Prevents Obesity- and Diabetes-Induced Muscle Atrophy through the Akt-Dependent Signaling Pathway

**Figure 3:**
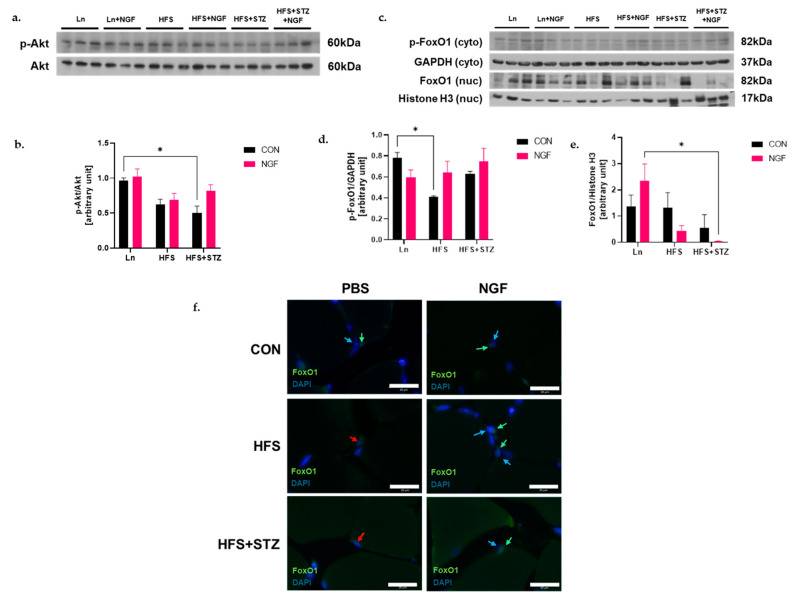
NGF prevents obesity- and diabetes-induced muscle atrophy through the Akt-dependent signaling pathway. (**a**) Representative immunoblots of p-Akt and Akt and densitometry of p-Akt/Akt ratio, * *p* < 0.05, Ln vs. HFS + STZ (**b**). (**c**) Representative immunoblots of the cytosolic fraction of p-FoxO1 and GAPDH and nuclear fraction of FoxO1 and Histone H3. (**d**) Densitometry of p-FoxO1 in cytosolic fraction. A significant decrease in phosphorylated FoxO1 is shown in HFS vs. Ln in the cytosolic fraction, * *p* < 0.05. (**e**) Densitometry of FoxO1 in nuclear fraction was significantly decreased in HFS + STZ + NGF vs. Ln+NGF, * *p* < 0.05. Data are shown as means ± SEM, *n* = 3 in each group. (**f**) FoxO1 translocation into the nucleus denoted by red arrows; FoxO1 is denoted by green arrows and nuclei is denoted by blue arrows; 40×; scale bar 20 μm; *n* = 3 in each group.

The PI3-kinase/Akt signaling pathway modulates muscle mass by inhibiting the translocation of the FoxO1 transcription factor into the nucleus [23]. In past studies, NGF enhanced the expression of Akt, thereby inhibiting the translocation of FoxO1 [24]. Thus, to investigate the effect of NGF in the Akt-FoxO1 signaling pathway in obese- and diabetes-induced muscle atrophy, the phosphorylation of Akt was evaluated in the gastrocnemius muscle tissues. As a result, the HFS + STZ group showed a significant decrease in phosphorylation levels of Akt while the HFS group only showed a trend (Figure 3b, * *p* < 0.05, HFS + STZ vs. Ln). This decreased level of phosphorylated Akt was improved in the HFS + STZ + NGF group (Figure 3b), although it did not reach significance. The cytosolic fraction of phosphorylated FoxO1 protein was significantly lower in the HFS group compared to the Ln control group (Figure 3d, * *p* < 0.05, HFS vs. Ln), which was alleviated by NGF treatment. To observe the localization of FoxO1 in HFS and HFS + STZ muscles, the level of FoxO1 in the nuclear fraction was analyzed. As shown in Figure 3e, the level of FoxO1 in the nuclear fraction showed a decreased trend in HFS + NGF and HFS + STZ mice compared to their non-treated counterparts. A significance was shown between Ln + NGF and HFS + STZ + NGF (Figure 3e, * *p* < 0.05, Ln + NGF vs. HFS + STZ + NGF). Immunofluorescent staining was performed to validate the translocation of FoxO1 into the nucleus. In both HFS and HFS + STZ groups, FoxO1 colocalized with the nucleus (red arrows, Figure 3f), and NGF treatment was shown to induce nuclear exclusion of FoxO1 (green and blue arrow to denote FoxO1 and nuclei, respectively, Figure 3f).

### 2.5. Levels of Ubiquitin Ligases and Autophagy Markers

**Figure 4:**
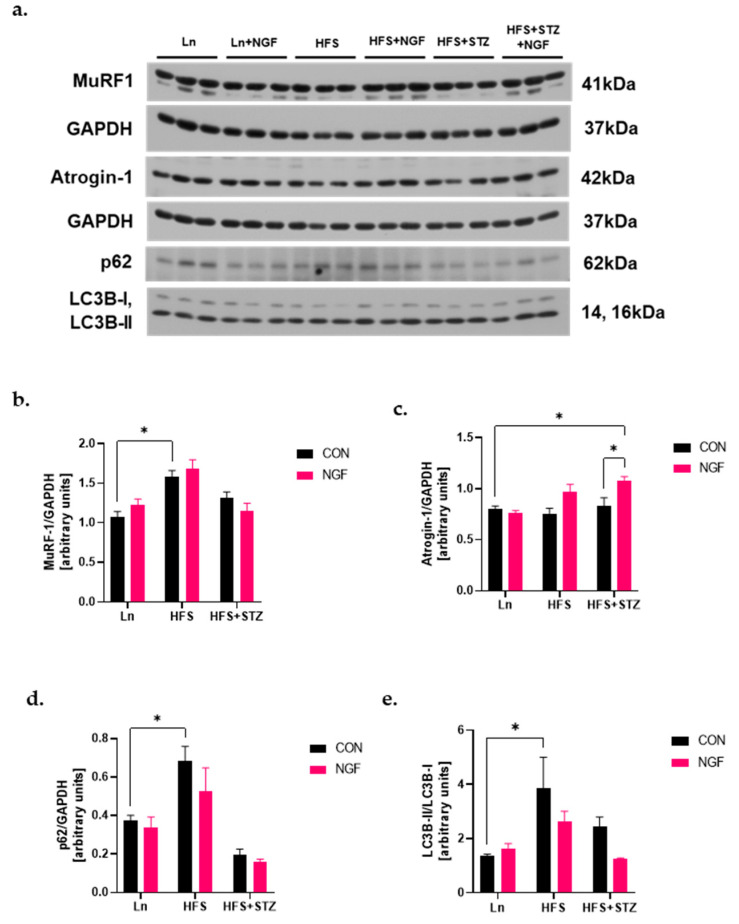
Levels of ubiquitin ligases and autophagy markers. (**a**) Representative immunoblots of MuRF1, Atogin-1, p62, and LC3B-I/II. (**b**) Densitometry of MuRF1. A significant increase was observed in HFS vs. Ln, * *p* < 0.05. (**c**) Densitometry of Atrogin-1. A significant increase was observed in HFS + STZ + NGF vs. Ln and HFS + STZ, * *p* < 0.05. (**d**) Densitometry of p62. A significant increase was observed in HFS vs. Ln, * *p* < 0.05. (**e**) Densitometry of LC3B-II/LC3B-I ratio. A significant increase was observed in HFS vs. Ln, * *p* < 0.05. Data are shown as means ± SEM, *n* = 3 in each group.

The transcriptional activity of FoxO1 is known to play a role in skeletal muscle atrophy through the expression of protein markers involved in the two major protein degradation systems [25]. Given that, we investigated the level of muscle-specific E3 ubiquitin ligases, muscle RING finger protein-1 (MuRF1) and Atrogin-1, and p62/SQSTM1 (referred to as p62 hereafter) and LC3B involved in the ubiquitin–proteasome system (UPS) and autophagy–lysosome pathway (ALP), respectively. The level of MuRF1 was significantly increased in the HFS group (Figure 4b, * *p* < 0.05, HFS vs. LN), but did not show a significant difference in the NGF-treated group. Atrogin-1 level was similar across groups, except that a significant increase in Atrogin-1 was observed in the HFS + STZ + NGF group (Figure 4c, * *p* < 0.05, HFS + STZ + NGF vs. Ln). The p62 level of the obese mice was significantly increased compared to the Ln group (Figure 4d, * *p* < 0.05, HFS vs. Ln). Although not significant, NGF-treated HFS and HFS + STZ groups show a decreased trend of p62 compared to their counterparts. Likewise, the ratio of LC3B-II/LC3B-I was increased in the HFS group compared to the Ln control, with a decreased trend in the NGF-treated groups (Figure 4e, * *p* < 0.05, HFS vs. Ln).

### 2.6. NGF Promotes Muscle Regeneration Capacity through Cyclin D1

**Figure 5:**
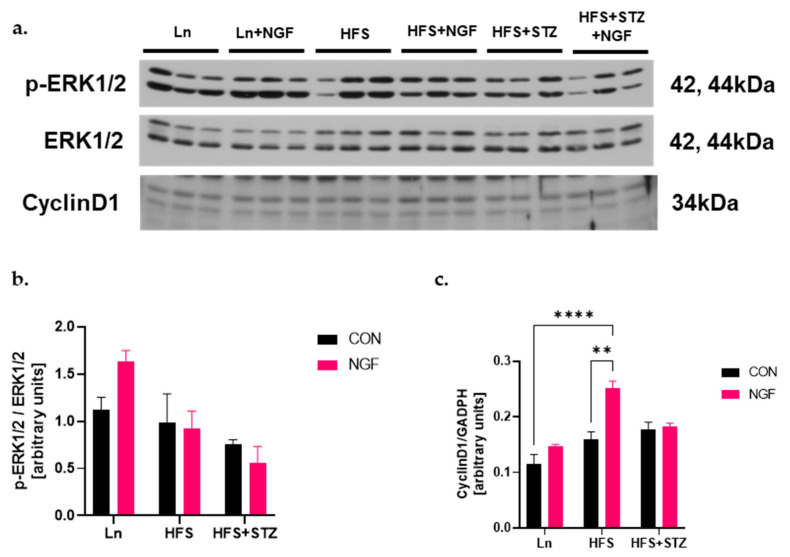
NGF promotes muscle regeneration capacity through cyclinD1. (**a**) Representative immunoblots of p-ERK1/2, ERK1/2, and cyclin D1. (**b**) Densitometry of p-ERK1/2 over ERK1/2 ratio. (**c**) Densitometry of cyclin D1. A significant increase was observed in HFS + NGF vs. HFS and Ln, ** *p* < 0.001 and **** *p* < 0.0001, respectively. Data are shown as means ± SEM, *n* = 3 in each group.

NGF promotes neuronal differentiation of PC12 cells by inducing cyclin D1 promoter, mRNA, and protein expression [26]. In contrast to myostatin, which induces G1 phase cell cycle arrest [27], cyclin D1 induces G1/S phase cell cycle progression [26]. Since NGF induction of the cyclin D1 promoter is Ras-dependent extracellular signal-regulated kinase (ERK)1/2-dependent, the level of ERK1/2 was examined through Western blotting. The levels of ERK1/2 were insignificant between the HFS and HFS + STZ groups compared to the Ln control group (Figure 5b). Cyclin D1 level in the HFS + NGF group was significantly higher than the non-treated counterpart (Figure 5c, * *p* < 0.05, HFS + NGF vs. HFS).

## 3. Discussion

While there is a significant awareness of the connection between obesity and T2DM with the risk of cardiovascular disease and other metabolic complications, less attention has been given to the impact of this metabolic state on skeletal muscle mass and quality. Thus, this study aimed to investigate the molecular mechanisms underlying skeletal muscle atrophy in obese and T2DM mice. First, this study analyzed the levels of markers involved in the synthesis and degradation pathways of skeletal muscle in obese and T2DM mice. We then analyzed the effect of NGF in these molecular pathways. As far as the authors know, this study is the first to link the impact of intranasal administration of NGF on skeletal muscle tissues in an animal model.

Under catabolic conditions, skeletal muscle atrophy occurs via two major protein degradation systems: UPS and ALP. The UPS proteolyzes short and soluble myoproteins through E1, E2, and E3 machinery [28]. Out of the vast pool of E3, Atrogin-1 and MuRF1 have been reported to be the major ubiquitin ligases contributing to muscle protein degradation [14], and knockout mice lacking either of these ligases exhibit reduced muscle atrophy [14]. The present study found a significantly increased level of MuRF1 in obese mice but not in T2DM mice. Moreover, the Atrogin-1 level was not elevated in these mice compared to the lean control mice. It is noteworthy that although NGF administration alleviated translocation of FoxO1 into the nucleus, which regulates transcription of both E3 ligases, the levels of the ligases exhibited an increasing trend compared to the non-treated counterparts. One possible explanation for the increase may be NGF binding to TrkA and p75NTR receptors, leading to subsequent activation of NF-κB transcription factors, which are known to trigger both MuRF1 and Atrogin-1 expression [14].

Macroautophagy, autophagy hereafter, plays a critical role in maintaining muscle homeostasis by removing protein aggregates and abnormal organelles that would otherwise cause muscle toxicity and dysfunctions [15]. However, excessive activation of ALP has been shown to result in muscle atrophy in past studies. p62 serves as a common adaptor protein for lysosomal and proteasomal substrates [14]. Meanwhile, microtubule-associated protein 1 light chain 3, LC3, serves as autophagosome markers that undergo proteasome-dependent degradation [14]. Conversion of LC3B-I to its active form, LC3B-II, is a crucial step in autophagy and considered a reliable marker of autophagosome formation [15]. Therefore, this study examined the levels of p62 and LC3B-II/LC3B-I ratio as markers of autophagic activity. The findings indicated that autophagy activity was higher in obese mice and decreased in the NGF-treated counterpart. A similar pattern was observed in the diabetic group, but the changes were not significant from the control group.

Traditionally, it has been believed that the UPS and ALP work by completely independent mechanisms; however, recent findings suggest that these two seemingly different machineries interact with one another [29]. Initial observations revealed that inhibition of one led to a compensatory upregulation of the other to maintain cellular homeostasis [29]. Thus, a possible speculation is that the increased levels of the E3 ligases observed in the NGF-treated obese and T2DM groups may be attributed to NGF’s capacity to attenuate the autophagic activity.

It is noteworthy that myostatin plays a role in regulating muscle UPS and ALP responses [30]. Consequently, we assessed the expression of myostatin at both the mRNA and protein levels. Our results show that mature myostatin protein levels were elevated in obese and T2DM mice, which is consistent with previous studies [31,32]. However, there was no significant difference found in myostatin at genetic levels between the obese and T2DM groups with the lean control group. Several explanations may account for these findings. First, we speculate that the decrease in mature myostatin protein may occur post-translationally because there is less of it in NGF-treated obese and diabetic mice, while there was no significant difference in the abundance of LAP forms of myostatin in all groups. Nevertheless, there is a possibility of feedback regulatory mechanism to a blockade in myostatin action by NGF. Indeed, there was a rise in myostatin mRNA level in the NGF-treated obese mice, contradicting the decreased level of mature myostatin protein.

Secondly, NGF may function through inhibiting myostatin’s cleavage from its complex form. Like in other TGF-β superfamilies, mature myostatin is released from the unprocessed precursor form through two discrete protease cleavage events. Initially, pro-myostatin is cleaved by proprotein convertases such as Furin or Proprotein Convertase Subtilisin/Kexin type 5 (PCSK5), which recognize a conserved RXXR site between the pro-domain and mature myostatin [33]. This cleavage results in an inactive latent complex with two pro-domains associated with the growth factor dimer. The second cleavage event occurs through a separate pro-domain cleavage by a protease from the BMP/Tolloid family, which releases the mature myostatin from the latent complex, allowing it to bind to its receptor and activate signaling [33]. Several myostatin antagonists, such as follistatin, have been studied in the past, which block the final cleavage of myostatin from its latent complex form. Our findings suggest that NGF may also be an inhibitor of myostatin cleavage, potentially mitigating muscle atrophy.

The mechanism of the increased myostatin mRNA expression in NGF-treated obese mice is unclear in the literature. It should be noted that while numerous studies observed an increased expression and activity of myostatin in several muscle atrophying conditions such as denervation [34], starvation [35], injury [36], and cancer cachexia [37], conflicting results have also been reported. For instance, mice with sepsis showed increased myostatin protein levels with decreased myostatin mRNA levels (rather than increased) [38]. Similarly, Carlson et al. [39] reported that seven days of hind leg unloading in mice resulted in muscle atrophy without changes in myostatin mRNA levels.

In addition to the regulation of myostatin expression, our study also investigated the effect of NGF on Akt/FoxO1 signaling pathways in the muscle. Our results showed significantly reduced levels of phosphorylated Akt in T2DM mice, while the NGF-treated counterpart showed an increased trend of phosphorylation of Akt. The phosphorylation of FoxO1 by phosphorylated Akt results in their nuclear export [24]. Indeed, we observed that NGF treatment alleviated the level of phosphorylated FoxO1 protein in the cytosolic fraction and decreased the level of FoxO1 in the nuclear fraction, although no significance was observed.

Lastly, our study investigated proteins involved in muscle regeneration and growth. The Ras-dependent ERK1/2 is the central player during cell proliferation. In normal cells, ERK1/2 is active, and its sustained expression is required for G1- to S-phase progression. Moreover, ERK1/2 is associated with induction of positive regulators of the cell cycle [40]. Likewise, cyclin D1 is a member of cyclin-dependent kinase (Cdk) family of serine/threonine kinases that are critical players in the progression of cell cycle [26]. The role of cyclin D1 in the cell cycle progression is complex, but it can promote G1-phase progression. Cyclin D is a downstream target of the Akt and ERK pathway. Moreover, NGF-induced ERK activity was shown to elevate the cyclin D1 level in PC12 cells [26] and human corneal epithelial cells [41]. Thus, we examined both ERK1/2 and cyclin D1 levels. Although we did not observe a significant difference in the phosphorylated ERK1/2 levels in obese or diabetic mice compared to lean control mice, cyclin D1 level was significantly increased in the NGF-treated obese mice. The increased level of cyclin D1, despite the insignificant level of activated ERK1/2, could indicate that the NGF-induced increase of cyclin D1 is favored via Akt pathway rather than ERK1/2 pathway. Additionally, myostatin is known to reduce cyclin D1 activity by promoting p21, a Cdk inhibitor, leading to the arrest of myoblasts in G1-phase of myoblast cell cycle [42]. Thus, the increase in cyclin D1 level supports the role of NGF in inhibiting the action of myostatin in obese mice.

This study has several limitations that need to be addressed. First, a gender difference in the markers of muscle hypertrophy and atrophy should be considered. For instance, studies have consistently shown that males express lower levels of myostatin than females [43], and certain catabolic conditions affect myostatin mRNA levels in a gender-dependent manner [44]. Second, male rodents are more susceptible to the diabetogenic effect of STZ than females due to hormonal differences between the genders [45], and this study did not explore the effect of STZ-induced diabetes in female models. Third, we did not measure the NGF concentration or mRNA levels in the gastrocnemius muscles. Although Frey et al. [46] reported that radiolabeled NGF administered via olfactory route can reach skeletal muscles, the amount of NGF concentration may vary between individual mice. Thus, we cannot guarantee that NGF acted similarly in all mice.

## 4. Materials and Methods

### 4.1. Animals

In brief, 5–6 week old male C57BL/6NHsd mice were purchased from Inotiv (Envigo) (West Lafayette, IN, USA) and were housed in an AAALAC-accredited facility. During the study period, the mice were housed in polycarbonate cages (3 mice per cage) in a temperature- and humidity-controlled room (74 +/− °F; 40–60% humidity) with a 12 h light–dark cycle. Initially, 11 or 12 mice were allocated to groups of six, and three mice were chosen to represent each group. All experimental procedures were approved by the Auburn University Institutional Animal Care and Use Committee aligning with the National Institutes of Health (NIH) guidelines. All efforts were made to minimize unnecessary suffering.

### 4.2. Diets

Animals were given either a standard chow diet (Teklad Global Rodent Diet 2018) with tap water or a Western diet composed of 45% fat (Cat. #5TJN, TestDiet, St. Louis, MO, USA) with sugar (42 g/L) added to the drinking water at a ratio of 55% fructose and 45% sucrose (HFS). Animals were given ad libitum access to food and water for 13 weeks after a one-week acclimation phase. Mice were randomly divided into six groups: control diet with PBS treatment (Ln), control diet with NGF treatment (Ln + NGF), HFS diet with PBS (HFS), HFS diet with NGF (HFS + NGF), HFS diet with STZ injection and PBS (HFS), and HFS diet with STZ injection and NGF (HFS + NGF).

### 4.3. Treatments

After 8 weeks on the diet, mice were intraperitoneally injected with streptozotocin (STZ) dissolved in 0.05 M citrate buffer pH 4.5 for three consecutive days at a dose of 40 mg/kg bodyweight to represent a T2DM model. One week after the final STZ injection, fasting blood glucose was measured by a FreeStyle FLASH glucometer and strips (Abott, Alameda, CA, USA). STZ-injected mice with a fasting blood glucose level higher than 200 mg/dl were considered diabetic. STZ-injected mice with a blood glucose level lower than 200 mg/dl were excluded from the study. After 9 weeks on diet, subgroups of mice were given intranasal injections of NGF dissolved in PBS at a concentration of 0.1 mg/mL, at 2 day intervals until the end of the study period. Each conscious mouse received an application of 5 μL drops in each nostril, altering the nostrils (left-right) with a lapse of 2 min between each administration.

### 4.4. Total Tissue Lysate Preparation

On the day of sacrifice, gastrocnemius tissues were isolated, washed with PBS, and stored in a −80 °C freezer. Frozen tissues were lysed with RIPA buffer (ThermoFisher Scientific, Rockford, IL, USA) using a hand-held homogenizer. Every 10 mg of tissue was homogenized with 100 μL of 1X RIPA buffer. The lysates were centrifuged at 12,000 rpm for 20 min at 4 °C. The supernatant lysates were collected, and protein concentration was analyzed using Pierce™ 660 nm Protein Assay Reagent (ThermoFisher Scientific, Rockford, IL, USA).

### 4.5. Tissue Subcellular Fractionation

The subcellular fractionation was modified from Dimauro et al. [47] Briefly, frozen gastrocnemius tissues were homogenized in STM buffer (250 mM sucrose, 50 mM Tris-HCl (pH 7.4), 5 mM MgCl2, DTT, and protease cocktail). Every 10 mg tissue was homogenized with 100 μL STM buffer. Samples were centrifuged at 800× *g* for 15 min at 4 °C. The supernatant was collected as cytosolic fraction. The pellet was resuspended in 500 mL STM buffer and centrifuged at 500× *g* for 15 min. The pellet was then resuspended in STM buffer and centrifuged at 1000× *g* for 15 min. The pellet was collected and resuspended in NET buffer (20 mM HEPES (pH 7.9), 1.5 mM MgCl2, 0.5 M NaCl, 0.2 mM EDTA, 20% glycerol, 1% Triton-X-100, and protease inhibitor cocktail) then incubated for 30 min in ice. The lysate was then centrifuged at 9000× *g* for 30 min at 4 °C. The supernatant was collected as the final nuclear fraction. Protein concentrations were determined using Pierce™ BCA Protein Assay Kit (ThermoFisher Scientific, Rockford, IL, USA).

### 4.6. Western Blot Analysis

The lysate samples were heated in sodium dodecyl sulfate-polyacrylamide gel electrophoresis (SDS-PAGE) sample buffer. Protein (20 μg) was separated on 8–12% polyacrylamide gels and then transferred onto polyvinylidene difluoride (PVDF) membranes (ThermoFisher Scientific, Rockford, IL, USA). The membranes were blocked for one hour in 5% non-fat milk at room temperature and then incubated with the following primary antibodies in 1:1000 or 1:2000 ratio at 4 °C overnight: myostatin (#sc-134345, Santa Cruz Biotechnology, Santa Cruz, CA, USA), p-Akt (#4060, Cell Signaling, Danvers, MA, USA), Akt (#9101, Cell Signaling), p-FoxO1 (#9464, Cell Signaling), FoxO1 (#2880, Cell Signaling), p-ERK1/2 (#9102, Cell Signaling), ERK1/2 (#9102, Cell Signaling), MuRF1 (#sc-398608, Santa Cruz Biotechnology), Atrogin-1 (#sc-166806, Santa Cruz Biotechnolgoy), LC3BI/II (#83506, Cell Signaling), p62 (#16177, Cell Signaling), Cyclin D1 (#sc-753, Santa Cruz Biotechnology), Histone H3 (#4499, Cell Signaling), and GAPDH (#MA5-15738, Invitrogen, Carlsbad, CA, USA). Equal loading of the protein was verified with GAPDH (myostatin, GAPDH (Figure 2b), p62 (Figure 4a), cyclin D1 (Figure 5a) were all processed in the same gel, so the GAPDH from Figure 2b was used to quantitate for densitometry) or histone H3 for the nucleus fractions. GAPDH and histone H3 were blotted on both cytosolic and nuclear fractions to assess purity of the samples (Supplemental Figure S1). Immunoblots were then incubated with horseradish peroxidase (HRP)-conjugated anti-mouse IgG (#ADI-SAB-100-J, Enzo Life Sciences, Farmingdale, NY, USA) or anti-rabbit IgG (#ADI-SAB-300-J) in 1:3000-1:7000 ratio at room temperature followed by a chemiluminescence detection protocol (Perkin Elmer Biosystems, Waltham, MA, USA). The proteins were quantified using ImageJ software, v1.54i (National Institutes of Health, USA). Densitometry analyses represent mean of three biological replicates.

### 4.7. RT-PCR

Total RNA from the frozen gastrocnemius muscle tissue samples was isolated using TRIzol reagent (Thermo Fisher Scientific, Rockford, IL, USA) and RNeasy Micro kit (QIAGEN, Venlo, The Netherlands) according to the manufacturer’s instructions. Genomic DNA was eliminated with RNase-Free DNase set (QIAGEN). Isolated RNA (200 ng) was reverse transcribed into complementary DNA (cDNA) using iScript cDNA synthesis kit (#1708840, Biorad, Hercules, CA, USA). RT-PCR was performed using Prime Time Gene master mix (#1055770, Integrated DNA Technologies, San Diego, CA, USA) and predesigned PCR primers (Integrated DNA Technologies) according to the manufacturer’s instructions. Each PCR reaction contained 2 μL of the sample cDNA (produced from 1 μg total RNA). The following predesigned PCR primers were obtained from Integrated DNA Technologies: Mstn (Mn.PT.58.13573446) and Actb (Mm.PT.39a.22214843.g). Primer and probe sequences are shown in Table 1. All primer and probe assays were used at a concentration of 500 nM. RT-PCR was performed using the Quantstudio 3 Real-Time PCR System (Thermo Fisher Scientific, Waltham, MA, USA). The relative expression levels between groups were compared and average values from two technical replicates were calculated.

### 4.8. Muscle Histology

After harvest, gastrocnemius muscle tissue samples were fixed in fixative consisting of 4% paraformaldehyde for 48 h. The tissues were then washed in PBS and stored in 70% ethanol at 4 °C. Tissues were cut into smaller pieces to be processed in Leica TP1020 automatic tissue processor (Leica Biosystems, Deer Park, IL, USA) and subsequently embedded into paraffin blocks (HistoCore Arcadia C, Leica Biosystems, Deer Park, IL, USA). The paraffin-embedded tissue blocks were sliced into 5-μm-thick cross-sections using HistoCore Rotary Microtome (Leica Biosystems, Deer Park, IL, USA).

### 4.9. Hematoxylin and Eosin Staining

Hematoxylin and Eosin (H&E) staining was performed in accordance with conventional methods. In brief, tissue slices were deparaffinized and rehydrated, after which they were washed and treated with hematoxylin for nuclei staining followed by eosin for cytosolic staining. After applying Permount mounting reagent, images were obtained using a microscope (Revolve, Echo, San Diego, CA, USA).

### 4.10. BODIPY^TM^ Staining

After the deparaffination and rehydration process, antigens were unmasked by steaming in Antigen Retrieval Citra, pH-6.0 (#HK086-9K, BioGenes, Cupertino, CA, USA) for 10 min. After washing in PBS, sections were incubated in 0.1 mg/mL BODIPY^TM^ 492/515 (#D3922, Invitrogen) in DMSO for 30 min at room temperature. Sections were washed in PBS and mounted with ProLong^TM^ Gold Antifade Mountant with DAPI for nuclear staining (#P36931, Invitrogen). All stained sections were viewed with a fluorescence microscope (Revolve, Echo, San Diego, CA, USA).

### 4.11. Immunofluorescence Staining

After the deparaffination and rehydration process, tissue sections were steamed in Antigen Retrieval Citra, pH-6.0 (#HK086-9K, BioGenex, Fremont, CA, USA) for 20 min. After several washes, PowerBlock (#HK085-5K, BioGenex, Fremont, CA, USA) was applied to each section according to the product directions. The sections were then incubated in the anti-FoxO1 (#2880, Cell Signaling) in 1:100 ratio at 4 °C overnight. After several washes, sections were incubated in the anti-rabbit secondary antibody (#A11008, Invitrogen) in 1:20 ratio at room temperature for one hour. Sections were then washed with PBS and mounted with ProLong^TM^ Gold Antifade Mountant with DAPI for nuclear staining (#P36931, Invitrogen). All stained sections were viewed with a fluorescence microscope (Revolve, Echo, San Diego, CA, USA).

### 4.12. Statistical Analysis

All values were given as the mean ± standard error of mean (SEM). Two-way analysis of variance (ANOVA) combined with Tukey’s test was used for multiple comparisons (GraphPad Prism, San Diego, CA, USA). *p* < 0.05 was considered as statistically significant value.

## 5. Conclusions

**Figure 6:**
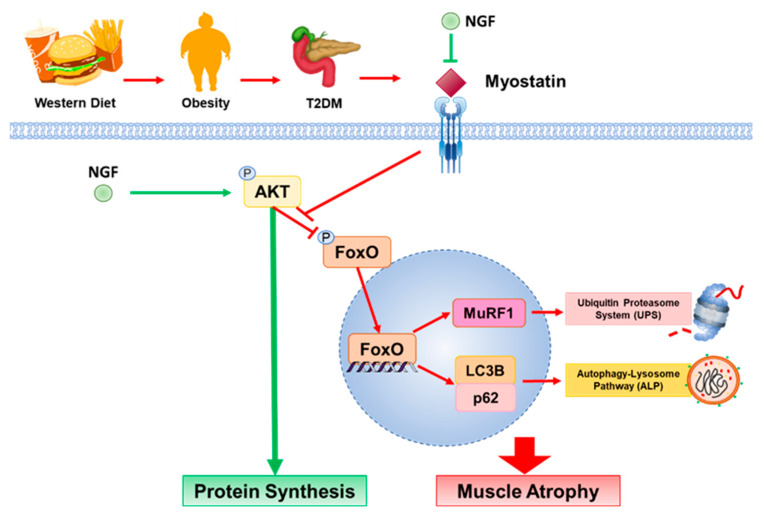
Diagram showing the effect of obesity and T2DM on the molecular pathways leading to skeletal muscle atrophy and involvement of NGF. An increased level of myostatin leads to the translocation of FoxO1 transcription factor, which induces muscle degeneration by increasing MuRF1, p62, and LC3BII. NGF inhibits the action of myostatin and enhances phosphorylation of Akt protein which then inhibits the translocation of FoxO1 into the nucleus. Moreover, NGF promotes muscle cell regeneration by increasing the level of cyclin D1. Arrows represent stimulation; blocked lines represent inhibition.

In summary, this study demonstrated that obesity and T2DM are associated with muscle atrophy by upregulating UPS and ALP while downregulating protein synthesis, resulting in muscle fiber shrinkage. Interestingly, the markers of muscle atrophy displayed disparate trends in obese and T2DM mice, as obese mice expressed higher levels of these markers than T2DM mice. These findings imply a multifaceted mechanism underlying muscle atrophy influenced by distinct metabolic conditions. Additionally, we observed that NGF has beneficial effect on preventing autophagy by regulating the processing of myostatin and inhibiting the nuclear translocation of FoxO1 transcription factor, thereby preventing muscle degradation (Figure 6). Moreover, NGF was shown to enhance the level of cyclinD1 via the Akt signaling pathway, which could potentially enhance muscle regeneration capacity. These findings shed light on the mechanisms underlying muscle atrophy caused by obesity and T2DM and suggest that NGF could be a potential therapeutic agent for preventing muscle atrophy in these conditions.

## Figures and Tables

**Table 1:** Primer sequences.

Gene	Primer Seq (5′–3′)	RefSeq
*Mstn*	FW: GCCATGATCTTGCTGTAACCT RV: CAGTCAAGCCCAAAGTCTCT /56-FAM/TCAGCCCAT/ZEN/CTTCTCCTGGTCCT/3IABkFQ/	NM_010834
*Actb*	FW: GATTACTGCTCTGGCTCCTAG RV: GACTCATCGTACTCCTGCTTG /5SUN/CTGGCCTCA/ZEN/CTGTCCACCTTCC/3IABkFQ/	NM_007393

## Data Availability

Data is contained within the article or Supplementary Material.

## Supplementary Materials

The following supporting information can be downloaded at: https://www.mdpi.com/article/10.3390/ijms25084307/s1.
