# Supplementary material for: Targeting Molecular Mechanisms of Obesity- and Type 2 Diabetes Mellitus-Induced Skeletal Muscle Atrophy with Nerve Growth Factor
Source: Int J Mol Sci. 2024 Apr 13;25(8):4307. doi: 10.3390/ijms25084307 (PMC11050157; doi:10.3390/ijms25084307)
Supplement: Supplementary file 1 [file ijms-25-04307-s001.zip › ijms-2895505-supplementary.pdf]

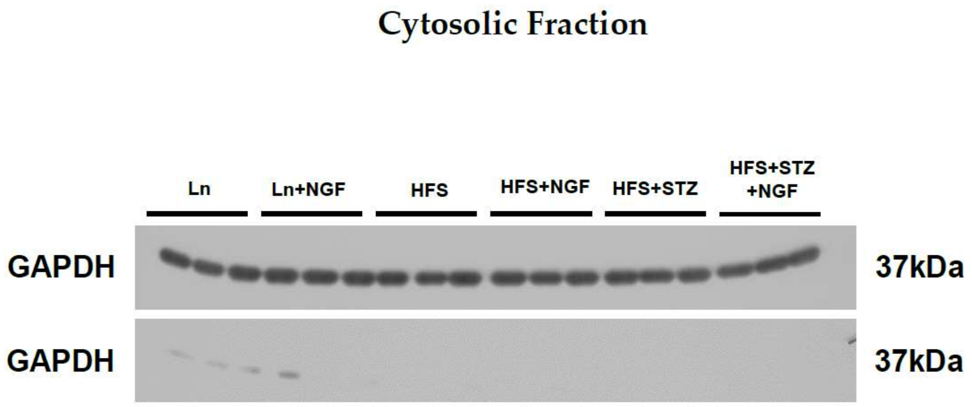


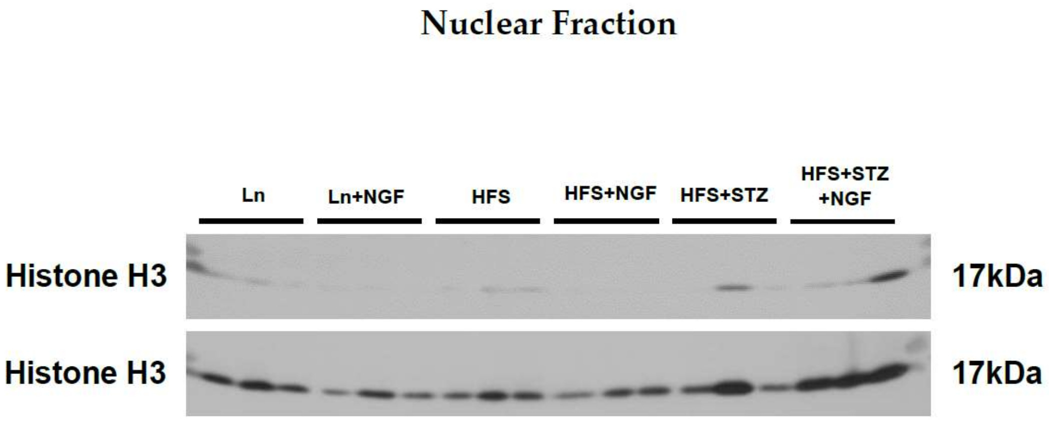


**Figure S1**. Western blot analysis of cytosolic and nuclear fractions from gastrocnemius muscle tissue lysate. Markers of cytosolic fraction (GAPDH) and nuclear fraction (Histone H3) were used to control fractionation quality.
